# Increased O-GlcNAcylation of c-Myc Promotes Pre-B Cell Proliferation

**DOI:** 10.3390/cells9010158

**Published:** 2020-01-08

**Authors:** Da Hee Lee, Na Eun Kwon, Won-Ji Lee, Moo-Seung Lee, Doo-Jin Kim, Ji Hyung Kim, Sung-Kyun Park

**Affiliations:** 1 Infectious Disease Research Center, Korea Research Institute of Bioscience & Biotechnology (KRIBB), 125 Gwahak-ro, Daejeon 34141, Korea; remake91@kribb.re.kr (D.H.L.); gyeoal@naver.com (N.E.K.); golddj@kribb.re.kr (D.-J.K.); kzh81@kribb.re.kr (J.H.K.); 2 Department of Stem Cell and Regenerative Biotechnology, Konkuk Institute of Technology, Konkuk University, Seoul 05029, Korea; dnjs303@konkuk.ac.kr; 3 Environmental Diseases Research Center, Korea Research Institute of Bioscience & Biotechnology (KRIBB), 125 Gwahak-ro, Daejeon 34141, Korea; msl031000@kribb.re.kr

**Keywords:** O-GlcNAcylation, pre-B cell, proliferation, c-Myc

## Abstract

O-linked β-N-acetylglucosamine (O-GlcNAc) modification regulates the activity of hundreds of nucleocytoplasmic proteins involved in a wide variety of cellular processes, such as gene expression, signaling, and cell growth; however, the mechanism underlying the regulation of B cell development and function by O-GlcNAcylation remains largely unknown. Here, we demonstrate that changes in cellular O-GlcNAc levels significantly affected the growth of pre-B cells, which rapidly proliferate to allow expansion of functional clones that express successfully rearranged heavy chains at the pro-B stage during early B cell development. In our study, the overall O-GlcNAc levels in these proliferative pre-B cells, which are linked to the glucose uptake rate, were highly induced when compared with those in pro-B cells. Thus, pharmacologically, genetically, or nutritionally, inhibition of O-GlcNAcylation in pre-B cells markedly downregulated c-Myc expression, resulting in cell cycle arrest via blockade of cyclin expression. Importantly, the population of B cells after the pro-B cell stage in mouse bone marrow was severely impaired by the administration of an O-GlcNAc inhibitor. These results strongly suggest that O-GlcNAcylation-dependent expression of c-Myc represents a new regulatory component of pre-B cell proliferation, as well as a potential therapeutic target for the treatment of pre-B cell-derived leukemia.

## 1. Introduction

During the course of B cell development, immunoglobulin (Ig) genes become sequentially activated by the regulation of V(D)J recombination mediated by recombination activating gene (RAG)-encoded recombinases and nonhomologous end-joining proteins [1]. The functionally rearranged Ig-heavy chains in pro-B cells can be assembled with surrogate light chains (SLCs) encoded by VpreB and λ5 to express pre-B cell receptor (pre-BCR) on the membrane surface, which indicates successful differentiation from the pro-B to the pre-B cell stage [2]. Once pre-BCR is adequately expressed, the pre-BCR-expressing large pre-B cells are transiently induced to rapidly proliferate and expand their functional clones. This phenomenon is a critical checkpoint during early B cell development [1,2]. In previous reports, autonomous signals of pre-BCRs coupled with the signal-transducing heterodimer Igα/Igβ primarily stimulated cell proliferation, with cells undergoing approximately four to six rounds of division [2,3]. Additionally, the Wnt (wingless-related mouse mammary tumor virus integration site)/β-catenin signaling pathway is involved in pre-BCR-mediated cell proliferation via regulation of Runt-related transcription factor (RUNX)/core-binding factor (CBF) β-targeted SLC expression bridged by charged amino acid-rich leucine zipper-1 (Crlz-1) [4,5]. Moreover, signaling from interleukin (IL)-7 receptor (IL-7R) on pre-B cells and that interact with IL-7-producing stromal cells in the bone marrow can influence pre-B cell proliferation by activating signal transducer and activator of transcription 5 [6].

O-linked β-N-acetylglucosamine (O-GlcNAc) modification is an important posttranslational modification of nucleocytoplasmic proteins [7,8]. In the cell, glucose and glucosamine imported via a glucose transporter can be converted to uridine diphosphate (UDP)-GlcNAc through the hexosamine biosynthetic pathway (HBP) [8]. O-GlcNAc transferase (OGT) adds the sugar moiety UDP-GlcNAc to serine or threonine hydroxyl groups of its target proteins, and this modification can be reversed through the removal of GlcNAc from proteins by glycoside hydrolase O-linked β-N-acetylglucosaminidase (OGA) [9,10]. To date, >1500 proteins have been identified as potential targets of O-GlcNAc modification and participate in nearly all aspects of cellular processes, including gene expression, insulin response, glycolysis, and signaling [11,12]. O-GlcNAcylation can directly affect the function of target proteins through changes in the phosphorylation pattern, stability, cellular localization, binding affinity to target sites, or ability to interact with other proteins [12,13]. Indeed, sustained perturbation of O-GlcNAc homeostasis in response to altered nutritional availability or external stress conditions is widely linked to a number of human diseases, including diabetes, neurodegenerative diseases, and cardiovascular diseases [11,14,15,16,17]. In particular, O-GlcNAcylation is abnormally elevated in different types of human cancers, including breast, prostate, lung, and colon cancers, as well as chronic myeloid leukemia, with several O-GlcNAcylated proteins in cancer cells highly associated with rapid cell proliferation, as well as tumor metastasis [18,19,20,21,22].

Recent studies restrictively described the specific functions of O-GlcNAc-modified proteins in B cells. Golks et al. [23] showed that induced O-GlcNAcylation of nuclear factor-kappa B (NF-κB) and nuclear factor of activated T cells (NFAT) is required for T- and B-lymphocyte activation. Additionally, the critical interplay between O-GlcNAcylation and phosphorylation for lymphocyte-specific protein-1 (Lsp-1) during B cell activation was proposed as a novel regulatory mechanism to explain how B cells control survival or apoptotic fate after BCR cross-linking [24]. Moreover, genetically engineered mouse models in which *Ogt* can be deleted at differential stages of B cell development showed not only defective activation of BCR signaling but also significant disruption of B cell homeostasis by enhancing apoptosis of germinal center B cells and memory B cells, which eventually resulted in reduced production of antibodies following immunization [25]. These findings suggest that O-GlcNAcylation plays crucial roles in B cell activation; however, the detailed molecular mechanisms associated with the stage-specific functions of this particular protein modification during B cell development are only beginning to be elucidated.

In this study, we hypothesized that rapidly proliferating large pre-B cells are sensitive to changes in cellular O-GlcNAc levels, similar to acutely growing cancer cells. To test this hypothesis, we first showed that pre-BCR-expressing large pre-B cells are differentiated to consume more glucose than pro-B cells during early B cell development, as previously reported [26], which appeared to consequentially induce GlcNAcylation in these pre-B cells. However, under conditions of low O-GlcNAcylation following inhibition of OGT activity in pre-B cells, proliferation was severely restricted due to the decreased expression of c-Myc (Myc proto-oncogene), which is an O-GlcNAc target protein, as well as a classical regulator of the cell cycle [27,28,29]. Indeed, downregulated expression of c-Myc directly modified by O-GlcNAcylation resulted in cell cycle arrest via inhibition of E- and A-type cyclin expression. In addition to disrupted OGT activity by treatment with a chemical inhibitor, glucose deprivation, or OGT knockdown, during the culture of pre-B cells markedly diminished cell proliferation accompanied by reduced O-GlcNAc levels and c-Myc expression. Interestingly, decreased c-Myc expression under glucose depletion was rescued by the re-introduction of glucose or glucosamine in continuous culturing experiments, with this activity naturally linked to recovered proliferation. In contrast to the dynamic changes in c-Myc expression dependent on cellular O-GlcNAc levels, the activity of canonical molecules previously recognized as primary regulators of pre-B cell proliferation, including pre-BCR, IL-7R, and Wnt/β-catenin, were unaffected by O-GlcNAc changes. These results suggested that the induction of O-GlcNAcylation in large pre-B cells during early B cell development was essential for the rapid proliferation of functional pre-B cell clones according to the O-GlcNAc status of c-Myc.

## 2. Materials and Methods

### 2.1. Cell Cultures and Reagents

The Abelson virus-transformed mouse pre-B cell line PD36 [4] and the human myelogenous leukemia cell line, a monocytic THP-1 (ATCC, TIB-202), were maintained at 37 °C in RPMI1640 media supplemented with 10% heat-inactivated fetal bovine serum (FBS; Corning) and 1× Antibiotic-Antimycotic (ThermoFisher Scientific, Waltham, MA, USA, 15240112 ) in an atmosphere of 5% CO_2_ saturated with water. In the case of PD36, L-glutamine (2 mM), nonessential amino acids (0.1 mM), sodium pyruvate (1 mM), and 2-mercaptoethanol (50 μM) were additionally provided in the culture media. For the cell culture in glucose-depleted media, PD36 pre-B cells were firstly seeded in 0 or 10 mM glucose-containing media supplemented with 1% FBS and 1 mM sodium pyruvate [30] and incubated for 24 h. Then, cells in glucose-depleted media were re-seeded with 0, 5, or 10 mM glucose or 1 mM glucosamine and incubated for 48 h. The reagents used were: OSMI-1 (Cayman, Ann Arbor, MI, USA, 21894), Thiamet G (Sigma-Aldrich, SML0244), dimethyl sulfoxide (Sigma-Aldrich, St. Louis, MO, USA, 276855), D-(+)-Glucosamine hydrochloride (Sigma-Aldrich, G4875), Glucose-free RPMI1640 (ThermoFisher Scientific, 11879020), glucose solution (ThermoFisher Scientific, A2494001), and 10058-F4 (Sigma-Aldrich, F3680)

### 2.2. Isolation of Lymphocyte Cells

Total bone marrow cells isolated from 6- to 8-week-old female C57BL/6 mice (Koatech, Pyeongtaek, Korea) were treated with 1X Red blood cell lysis solution (Miltenyi Biotec, Bergisch Gladbach, NRW, Germany, 130-094-183) at room temperature (RT) for 10 min. After centrifugation, the pelleted cells resuspended in phosphate-buffered saline (PBS) containing 1X Antibiotic-Antimycotic were forced through a 40-μm cell strainer (Corning Inc, Corning, NY, USA, 352235) and then the total numbers of single-cell suspension were counted. To concentrate the total bone marrow cells, B220-expressing cells were collected by positive selection on MACS columns with B220 microbeads (Miltenyi Biotec, 130-049-501). The selected cells were incubated with anti-CD16/32 (BD Biosciences, San Jose, CA, USA, 553141) to block Fc receptors at 4 °C for 20 min and then stained with FITC-anti-CD43 (BD Biosciences, 553270) and APC-anti-B220 (BD Biosciences, 553092) for the isolation of pro-B cells or biotinylated-anti-pre-BCR (BD Biosciences, 551863) and FITC-streptavidin (BD Biosciences, 554060) for pre-BCR expressing pre-B cells at 4 °C for 20 min. After antibody staining, cells were washed twice with sorting buffer [0.5% bovine serum albumin (BSA) and 2 mM ethylenediaminetetraacetic acid (EDTA) in PBS] and processed for positive cell sorting by a FACS Aria cell sorter (BD Bioscience). In the case of primary cell culture, the sorted cells were maintained at 37 °C in IMDM GlutaMAX (ThermoFisher Scientific, 31980030) supplemented with 10% FBS and 1X Antibiotic-Antimycotic.

### 2.3. Glucose Uptake Assay

Glucose uptake was measured by Glucose Uptake-Glo^TM^ Assay (Promega, Madison, WI, USA, J1341) following the manufacturer’s instructions. Briefly, isolated pro-B and large pre-B cells (1 × 10^4^ cells) by FACS described above were incubated with 2-deoxyglucose (2DG) in PBS at RT for 20 min, allowing conversion of 2DG to 2DG-6-phosphate (2DG6P) in the same manner as glucose in the cells. After incubation, stop buffer as an acidic detergent solution was treated to lyse cells and neutralization buffer was added to neutralize the acid. Then, detection reagent [glucose-6-phosphate dehydrogenase, nicotinamide adenine dinucleotide phosphate (NADP+), reductase, luciferase, and pro-luciferin] was applied to measure the luciferase activity triggered depending on the rate of glucose uptake. In principle, glucose-6-phosphate dehydrogenase (G6PDH) oxidizes 2DG6P from cells to 6-phosphodeoxygluconate, reducing NADP+ to NADPH. The reductase uses NADPH to convert pro-luciferin to luciferin, and then luciferase produces a luminescent signal using luciferin. The luciferase activity was measured by a Centro XS3 LB 960 (Berthold Technologies, Bad Wildbad, Germany).

### 2.4. Cell Proliferation Assays

Cells were counted using a Countess II FL Automated Cell Counter (ThermoFisher Scientific, AMQAF1000) with trypan blue staining or a LUNA-FL^TM^ Cell counter (Logos Biosystems, Anyang-si, Gyeonggi-do, Korea, L20001) with dual fluorescence staining, acridine orange (live cell detection), and propidium iodide (dead cell detection), to check cell viability at each time point under various drug treatments. Counts were duplicated and averaged every time. WST-1 dye-based cell viability assays were performed using EZ-CYTOX (Daeil Lab Service, Guro-gu, Seoul, Korea, EZ-1000) following the manufacturer’s protocol. Briefly, 100 mL of cultured cells were transferred to a 96-well plate in triplicate for each sample and then treated with 10 mL of WST-1 reagent to each well for 2 h in the standard culture conditions as described above. After incubation, absorbance was measured at 450 nm using a SpectraMAX 190 Microplate Reader (Molecular Devices, manufacture, San Jose, CA, USA).

### 2.5. Cell Cycle Assays

The cell cycle was analyzed by an FITC-BrdU Flow Kit (BD biosciences, 559619) following the manufacturer’s instructions. Briefly, PD36 cells were cultured with BrdU, an analog of the DNA precursor thymidine, for 2 h to determine the proliferated cells containing newly synthesized DNA, and then BrdU-incorporated cells were stained with FITC-anti-BrdU antibodies. To assess the cell cycle phases, 7-amino-actinomycin D (7-AAD) that binds to DNA was also used to treat cells while treating with immunofluorescent-BrdU-antibodies. Two color-stained cells were analyzed for the frequency of cells having synthesized DNA as at S-phase in the total cell cycle positions by a GALLIOS Flow Cytometer (Beckman Coulter, Brea, CA, USA) and FlowJo software. As an alternative assay for the cell cycle, cells were stained with propidium iodide (PI) to quantify their total DNA contents. Briefly, THP-1 cells were fixed with 1% paraformaldehyde (BD Biosciences, 51-2354AK) on ice for 1 h and then permeabilized with 70% ethanol. Cells were then collected by centrifugation and treated with 200 μL of RNase A (ThermoFisher Scientific, 200 μg/mL in PBS) at 37 °C for 30 min, and stained with 25 μg/mL of PI (ThermoFisher Scientific) at RT for 30 min. After two washes with PBS, the DNA contents of cells stained with PI were analyzed by a GALLIOS Flow Cytometer (Beckman Coulter) and FlowJo software (version 10.6.1, FlowJo LLC, Ashland, OR, USA).

### 2.6. Cell Cytotoxicity Assay

The cell cytotoxicity of the O-GlcNAc drugs used in this paper were assessed using propidium iodide (ThermoFisher Scientific), which is membrane impermeant and therefore does not enter viable cells. Briefly, cells cultured in inhibitor-containing media were stained with 25 μg/mL of propidium iodide and then analyzed by a GALLIOS Flow Cytometer (Beckman Coulter) and FlowJo software. As a positive control, cells were fixed with 1% paraformaldehyde (BD Biosciences, 51-2354AK) on ice for 1 h and then permeabilized with 70% ethanol. For the lactose dehydrogenase (LDH)-based assay, a Pierce^TM^ LDH Cytotoxicity Assay Kit (ThermoFisher Scientific, 88953) was used following the manufacturer’s protocol. Briefly, an appropriate number of pre-B cells were cultured in a 12-well plate with chemical inhibitors or vehicle only for 48 h, and then supernatant after incubation by centrifugation was taken for the assay. Culture media without any cells was used as a negative control and supernatant from the same number of cells treated with 10 μg/mL of puromycin (InvivoGen, San Diego, CA, USA, ant-pr-1) for 5 h in order to intentionally induce cell damage, resulting in a ~35% death rate, was used as a positive control. In principle, dead or dying cells released a cytosolic enzyme like lactate dehydrogenase (LDH) to culture media, so then the LDH enzymatic response with the reaction mixture in the kit produced a red formazan, which could be measured by a spectrophotometer.

### 2.7. Western Blotting

Total proteins were obtained from cells by lysis using RIPA buffer (ThermoFisher Scientific, 89900) containing 1× protease inhibitor cocktail (GenDEPOT, Katy, TX, USA, p3100-010) and phosphatase inhibitor (GenDEPOT, p3200-001). The protein concentration was estimated using a Pierce^TM^ BCA protein assay kit (ThermoFisher Scientific, 23225) and an appropriate amount of proteins was mixed with 5X SDS-PAGE sample buffer (TransLab, Dong-gu, Daejeon, Korea, TLP-102.1). Then, prepared proteins were separated by NuPAGE^TM^ 4–20% Bis-Tris protein gels (ThermoFisher Scientific, NP0322PK2) or traditional Tris-Glycine SDS-PAGE gels and transferred to polyvinylidene fluoride (PVDF) membrane in iBlot^TM^ 2 Transfer stacks (ThermoFisher Scientific, IB23001) using an iBlot^®^ 2 Gel Transfer Device (ThermoFisher Scientific, IB21001). The membranes were blocked with TBS containing 5% BSA and then incubated with primary antibodies in TBS-T containing 5% BSA overnight at 4 °C, and then with secondary antibodies conjugated with HRP or IR (infrared) dye at RT for 1 h, followed by 5 TBST-washes and one TBS-wash. Detected protein bands were visualized through an Odyssey Fc Imaging System and quantified using ImageStudio software (LI-COR Bioscience, Lincoln, NE, USA). The following antibodies were used in this study: Anti-O-GlcNAc (CTD110.6; SantaCruz Biotechnology, Dallas, TX, USA, SC-59623 or RL2; ThermoFisher Scientific, MA1-072), OGT (MilliporeSigma, Burlington, MA, USA, O6264), OGA (MilliporeSigma, SAB4200267), c-Myc (Cell Signaling Technology, Danvers, MA, USA, #9402), β-catenin (Cell Signaling Technology, #9562), GSK3α/β (SantaCruz Biotechnology, sc-7291,Dallas, TX, DT, USA), phospho- GSK3β (SantaCruz Biotechnology, sc-373800), Igμ (Sigma-Aldrich, A4540), VpreB (SantaCruz Biotechnology, sc-514957), p27 (Cell Signaling, #2552), p21 (SantaCruz Biotechnology, sc-6246), Nup98 (SantaCruz Biotechnology, sc-74553), and β-actin-conjugated HRP (Cell Signaling Technology, #5125).

### 2.8. Pull-Down Assay with WGA Lectin and Immunoprecipitation

O-GlcNAc-attached proteins were isolated using a Glycoprotein Isolation Kit, WGA (ThermoFisher Scientific, 89805) following the manufacturer’s protocol. Briefly, PD36 pre-B cells (1 × 10^7^) cultured with an appropriate inhibitor were lysed in 140 μL of SDS lysis buffer (0.5% SDS, 50 mM Tris-HCl (pH 6.8), 1 mM EDTA, 1 mM DTT, 1× protease inhibitor cocktails) with incubation at 65 °C for 5 min, and then diluted with lysate using 560 μL of ice-cold correction buffer (1.25% NP-40, 0.625% sodium deoxycholate, 62.5 mM Tris-HCl (pH 8.0), 2.25 mM EDTA, 187.5 mM NaCl, 1× protease inhibitor cocktails), which was put through a QiaShredder (Qiagen, Hilden, Garmany, 79654) twice to rupture genomic DNA. While preparing the cellular extracts, 200 μL of WGA lectin resin (50% slurry) were washed three times with 1× Binding/Wash Buffer. In total, 400 μL of cellular extracts (400 μg) mixed with 100 μL of 5× Binding/Wash buffer were added to the pre-washed resin and then incubated for 30 min at room temperature with end-over-end mixing using a rotator. Following incubation, the resin captured with O-GlcNAcylated proteins by WGA lectin was washed 4 times with 1× Binding/Wash buffer and eluted by 2× SDS-PAGE sample buffer in RIPA buffer with heating and shaking at 95 °C for 10 min. In case of immunoprecipitation, an appropriate antibody (2 μg) was mixed with 40 μL of SureBeads protein A magnetic beads (Bio-Rad, Hercules, CA, USA, 161-4011) by incubation for 20 min at RT. Cellular extracts (~150 μg) were pre-cleared using an isotype-matched antibody and protein A magnetic beads at 4 °C for 1 h. Then, pre-cleared extracts were incubated with magnetized antibody-beads complex at 4 °C by overnight rotation. After incubation, captured proteins by antibody-magnetic beads were washed 5 times with PBS-T (PBS plus 0.1% Tween-20) and eluted by 1× SDS-PAGE sample buffer in RIPA buffer with heating at 95 °C for 10 min.

### 2.9. Quantitative RT-PCR

Total RNAs were prepared from cells harvested from incubation under various conditions using TRIzol reagent (Molecular Research Center, Cincinnati, OH, USA, TR118) and followed by treatment with RQ1 DNase (Promega) following the manufacturer’s protocol. The resulting RNA samples were reverse-transcribed to synthesize cDNA using an iScript^TM^ cDNA Synthesis Kit (Bio-Rad, 1708890), followed by real-time reactions using iTaq^TM^ Universal SYBR^®^ Green Supermix (Bio-Rad, 1725121) by a LightCycler^®^ 96 system (Roche, Basel, Switzerland). The ΔΔCq method was used to calculate the relative gene expression. The nucleotide sequences of the primers used in the real-time reaction are given in Supplementary Table S1.

### 2.10. Plasmid Constructs

The wild-type (WT) cDNA clone for mouse c-Myc in pCMV6-Entry vector was purchased from OriGene (Rockville, MD, USA, MR227361), which has a FLAG-tag at the C-terminal. The point mutation in the specific amino acid site of WT c-Myc was generated using a Phusion Site-Directed Mutagenesis Kit (ThermFisher Scientific, F-541) following the manufacturer’s protocol. Briefly, the WT c-Myc construct was amplified with two phosphorylated primers with the desired mutation, which are designed to anneal back to back to the plasmid. The PCR products were digested by DpnI enzyme to remove Dam-methylated parental plasmid DNA and then circularized using T4 DNA ligase. The resulting reaction mixture was proceeded for heat-shock transformation to competent *Escherichia coli* NEB^®^-5-alpha (NEB, Ipswich, MA, USA, C2987I). The substituted sequences were confirmed by DNA sequencing with T7 primer. The nucleotide sequences of the primers used in this procedure are given in Supplementary Table S1.

### 2.11. Transfection

Transient transfection for overexpression of c-Myc cDNA and its mutant constructs was performed by a Neon^TM^ Transfection System (ThermoFisher Scientific, MPK5000) following the manufacturer’s protocol. Briefly, 3 × 10^5^ cells of PD36 were washed by PBS and then collected by centrifugation. Pelleted cells were resuspended with a premixed solution of 11.25 μL of Neon resuspension buffer R plus 1.25 μL of plasmid DNA (0.25 pmole). In total, 10 μL of the cell–DNA mixture was taken into a 10-μL Neon tip using a Neon pipette, which was then put into a Neon tube containing 3 mL of cold Neon electrolytic buffer E on the Neon pipette station. The cell–DNA mixture was electrically pulsed twice at 1500 V with a pulse width of 20 ms and transferred immediately into a 6-well plate containing pre-warmed culture media. In case of small interfering RNA (siRNA) transfection, 1 × 10⁶ cells of PD36 were pelleted and resuspended with a premixed solution of 100 μL of Neon resuspension buffer R plus 10 μL of 10 μM siRNA. In total, 100 μL of the cell–DNA mixture was taken into a 100-μL Neon tip using Neon pipette, and then the cell–DNA mixture was electrically pulsed twice at 1450 V with a pulse width of 25 ms and transferred immediately into a 100-mm culture dish containing glucose-depleted media with 2 mM glucose. The following Silencer^TM^ Select Pre-Designed siRNAs were used in this study: Negative Control No.1 (ThermoFisher Scientific, 4390843) and Mouse Ogt (ThermoFisher Scientific, 4390771).

### 2.12. ELISA

For the detection of OGT or O-GlcNAc, 96-well plates (Corning) were coated with lysates from pre-B cells transfected by siOGT or siCONTROL, serially diluted in PBS, at 4 °C overnight. The plates were washed thrice with PBS-T (0.05% Tween 20 in 1× PBS) and incubated in blocking buffer (0.1% Tween 20 and 1% BSA in PBS) at RT for 2 h, followed by PBS-T-washes three times. The plates were washed five times with PBS-T and treated with a primary antibody, anti-OGT, or anti-O-GlcNAc (CTD110.6), diluted 1:1000 in an assay buffer (0.05% Tween 20 and 0.05% BSA in PBS) at RT for 2 h, followed by PBS-T washes five times. Then, detecting antibody (HRP-conjugated anti-mouse IgG or IgM) was diluted 1:5000 in an assay buffer at RT for 1 h. For the detection of c-Myc, 96-well plates were initially coated with 1 μg/mL of capture antibody, anti-c-Myc, at 4 °C overnight, and then cell lysates were applied to the plate after blocking. HRP-conjugated anti-Myc antibody was used as a detecting antibody. After five washes with PBS-T post treatment of each detecting antibody, TMB Substrate Solution (BioLegend, San Diego, CA, USA, 77247 & 77248) was added to the plates. After an appropriate time of incubation, the reaction was stopped by adding 2 N sulfuric acid (MilliporeSigma). The plates were finally read at 450 nm using a SpectraMAX 190 Microplate Reader (Molecular Devices).

### 2.13. Treatment of OGT Inhibitor to Mice and Flow Cytometry Analysis

In total, 1 mg of OSMI-1 was solubilized in 100 mL of buffer containing 4.5% DMSO and 5% Tween-80 (MilliporeSigma, P8074) in water. Then, 100 mL of OSMI-1 solution was intraperitoneally injected every 24 h for a week to 5-week-old female BALB/c mice (Koatech) immunized once with 50 μg of NP-CGG (Biosearch, Novato, CA, USA, N-5055C-5) in 50 mL of PBS with 50 mL of Imject^®^ Alum (ThermoFisher, 77161) on the 2nd day. At the next day of final injection, total bone marrow cells were extracted from mice, and FACS analyses was proceeded to. Cells were incubated with anti-CD16/32 to block Fc receptors at 4 °C for 20 min and then stained with PerCP-Cy5.5-anti-CD43 and APC-anti-B220 at 4 °C for 20 min. After two washes with PBS, cell population analyses were performed by a GALLIOS Flow Cytometer (Beckman Coulter) and FlowJo software.

### 2.14. Ethics Statement in Animal Study

All mice were kept under a specific pathogen-free facility in the Korea Research Institute of Bioscience and Biotechnology (KRIBB, Daejeon, Korea), and all animal studies were performed in accordance with protocols approved by the Animal Experiments Ethics Committee at KRIBB (KRIBB-AEC-19194, 02-09-2019).

### 2.15. Statistical Analyses

A two-tailed Student’s t test in Microsoft Excel was used to determine the statistical significance. Comparison groups are indicated in each figure legend. Significance is annotated as * *p* ≤ 0.05, ** *p* ≤ 0.01, and *** *p* ≤ 0.001.

## 3. Results

### 3.1. Pre-BCR-Expressing Large Pre-B Cells Sorted from Mouse Bone Marrow Show Highly Induced Cellular O-GlcNAc Levels Relative to Pro-B Cells

**Figure 1:**
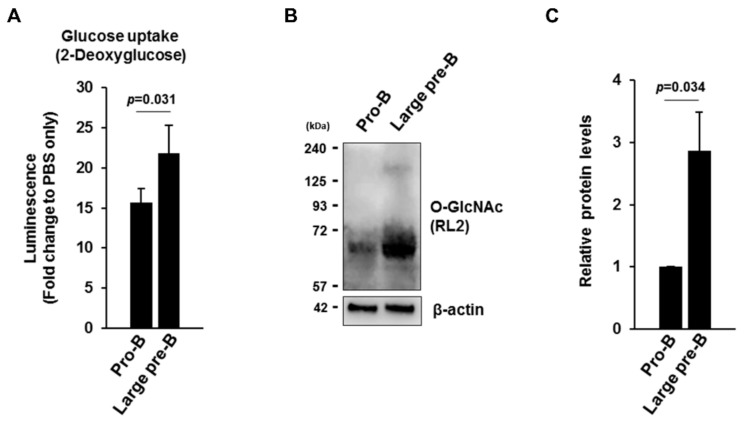
Glucose uptake and cellular O-GlcNAc levels are upregulated at the large pre-B cell stage relative to those in pro-B cells. (**A**) Glucose uptake assay using pro-B cells (B220^+^IgM^−^CD43^+^) and large pre-B cells (B220^+^Pre-BCR^+^) sorted from mouse bone marrow (BM). Luminescence was measured as an indicator of the glucose consumption rate in 1 × 10^4^ cells. Data are represented as mean ± SD (*n* = 3) normalized using controls treated with PBS only without any cells. (**B**) Representative western blot showing bulk O-GlcNAcylation in pro-B cells and large pre-B cells. (**C**) Quantification of data from western blot results in (**B**). Data are represented as mean ± SD (*n* = 3) normalized against β-actin, which was used as a loading control. (**A**–**C**) Pooled BM cells from eight or four mice for each experiment were used to isolate pro-B or large pre-B cells, respectively.

Because cellular metabolism is dynamically altered during the transition from pro-B to pre-B cells during early B cell development [26], we hypothesized that rapidly proliferating large pre-B cells might consume additional glucose to support their active cell metabolism and subsequently upregulate cellular O-GlcNAc levels. To test this hypothesis, we first compared glucose uptake rates, as well as overall O-GlcNAc levels, between pro-B (CD43 + IgM-) and large pre-B cells (pre-BCR+) isolated by fluorescence-activated cell sorting (FACS) from mouse bone marrow after the enrichment of B220+ cells by magnetic-activated cell sorting (MACS) (Supplementary Figure S1A–C). As expected, large pre-B cells sorted by pre-BCR expression as a marker showed higher levels of glucose uptake than pro-B cells (Figure 1A), with cellular O-GlcNAc levels upregulated accordingly in these pre-BCR-expressing cells (Figure 1B,C). These results suggest that increased glucose uptake by large pre-B cells during early B cell development presumably promotes higher production of UDP-GlcNAc, an OGT substrate [31], thereby elevating protein O-GlcNAcylation in these pre-B cells.

### 3.2. Decreased O-GlcNAcylation Significantly Restricts Pre-B Cell Proliferation

**Figure 2:**
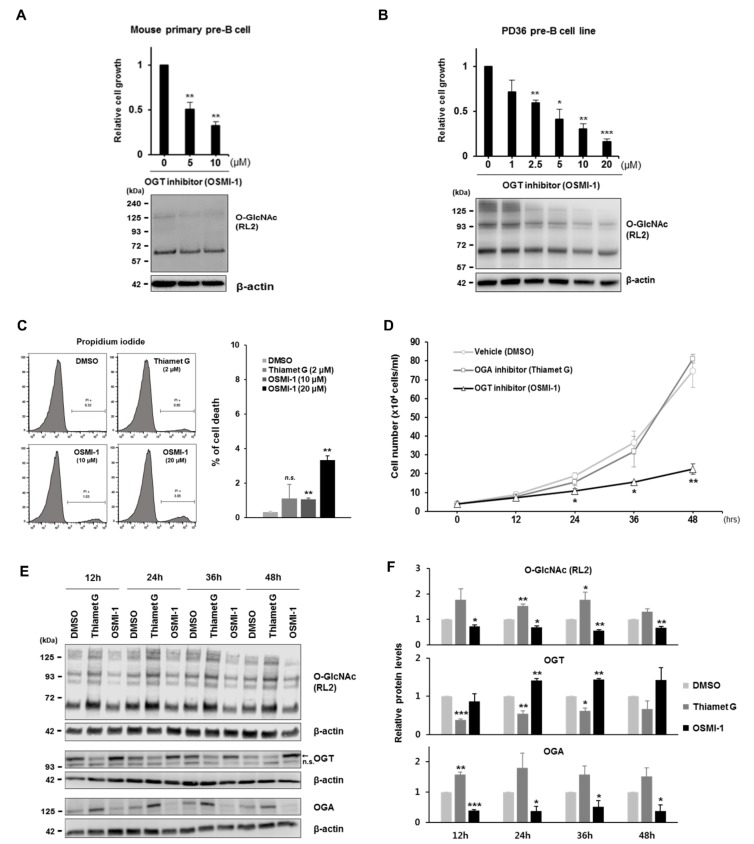
Proliferation of large pre-B cells expressing pre-BCR is significantly decreased by inhibition of O-GlcNAcylation. (**A**,**B**) Relative cell number after a 48-h culture using different doses of OSMI-1 (OGT inhibitor) and normalized against DMSO (vehicle control; 0 μM). Data are represented as mean ± SD (*n* = 3). Comparison of OSMI-1 treatment at each dose with DMSO treatment. Representative western blot (bottom) to monitor decreased O-GlcNAcylation. (**A**) Mouse primary pre-B cells were sorted using the pre-BCR expression as a marker. (**B**) Pre-BCR-expressing pre-B cells, PD36. (**C**) Representative plot showing data from flow cytometric analysis of cell death in PD36 pre-B cells stained with propidium iodide at 48 h post-treatment with inhibitors [Thiamet G (OGA inhibitor; 2 μM, final) and OSMI-1 (10 or 20 μM, final)] (left). Quantification of the percentage of propidium iodide-positive cells as dead cells (right). Data are represented as mean ± SD (*n* = 3). Comparison of each treatment with DMSO-treated control. n.s. = not significant. (**D**) Cell growth of PD36 pre-B cells for 48 h following inhibitor treatment [Thiamet G (2 μM final) and OSMI-1 (10 μM final)]. The growth curve was generated by counting cells every 12 h. Data are represented as mean ± SD (*n* = 3). Comparison of OSMI-1 treatment with that of the DMSO control at each time point. (**E**) Representative western blot to monitor changes in O-GlcNAcylation, OGT, and OGA levels at each time point. (**F**) Quantification of data from western blot results in (**E**). Data are represented as mean ± SD (*n* = 3) normalized using β-actin as a loading control. Comparisons were performed using the DMSO control at each time point. (**A**–**D**,**F**) Significance is annotated as * *p* ≤ 0.05, ** *p* ≤ 0.01, and *** *p* ≤ 0.001.

Because induced O-GlcNAcylation is associated with increased cell proliferation, and given that large pre-B cells grow rapidly to expand their functional clones during early B cell development, we anticipated that elevated O-GlcNAcylation levels in large pre-B cells (Figure 1) might accelerate cell proliferation. To investigate this possibility, we determined whether pre-B cell proliferation is altered by changes in cellular O-GlcNAc levels. After exposure of pre-BCR-expressing pre-B cells to OSMI-1, a cell-permeable inhibitor of OGT [32,33], O-GlcNAc modification was effectively inhibited, and subsequent cell proliferation was markedly decreased in a dose-dependent manner, as expected (Figure 2A,B). Significantly, this regulatory effect of O-GlcNAc inhibition in cell proliferation was verified not only in the pre-BCR-expressing cell line, PD36, but also in primary pre-B cells sorted from mouse bone marrow using pre-BCR expression as a marker (Supplementary Figure S1C). Next, cell growth rates were determined again time-dependently, with 10 uM of OSMI-1 and 2 uM of OGA inhibitor Thiamet G, which were found to have little cytotoxicity in PI or LDH-based cell viability assays (Figure 2C, Supplementary Figure S2A). As a result, effective suppression of O-GlcNAcylation by OSMI-1 treatment was continued for up to 48 h and under these conditions, cell growth was inhibited by 25% or less compared to the control (Figure 2D–F, Supplementary Figure S2B). However, treatment with the OGA inhibitor, which increases O-GlcNAcylation [33,34], showed no effect on the growth rate of pre-B cells (Figure 2D–F). This assumes that O-GlcNAc levels in these pre-B cells were already sufficiently saturated to induce proliferation, such that further induction of O-GlcNAcylation would probably no longer enhanced cell growth. Contrary to these findings in pre-B cells, the growth rate of monocytic THP-1 cells was unaffected by changes in O-GlcNAc levels when altered by both inhibitor treatments (Supplementary Figure S3A,B).

Of particular interest in the field of O-GlcNAc biology, we observed dynamic changes in OGT and OGA levels in a direction toward recovery from altered O-GlcNAcylation caused by inhibitor treatment (Figure 2E,F). Indeed, OGT or OGA protein levels increased or decreased, respectively, upon inhibition of OGT activity and vice versa under OGA inhibition. This implies that not only the inhibitors were effective, but the compensatory mechanism between OGT and OGA to maintain O-GlcNAc homeostasis, previously reported in several different cell systems, was similarly well preserved in pre-B cells [32,33,35].

### 3.3. Decreased O-GlcNAcylation in Pre-B Cells Downregulates c-Myc Expression

**Figure 3:**
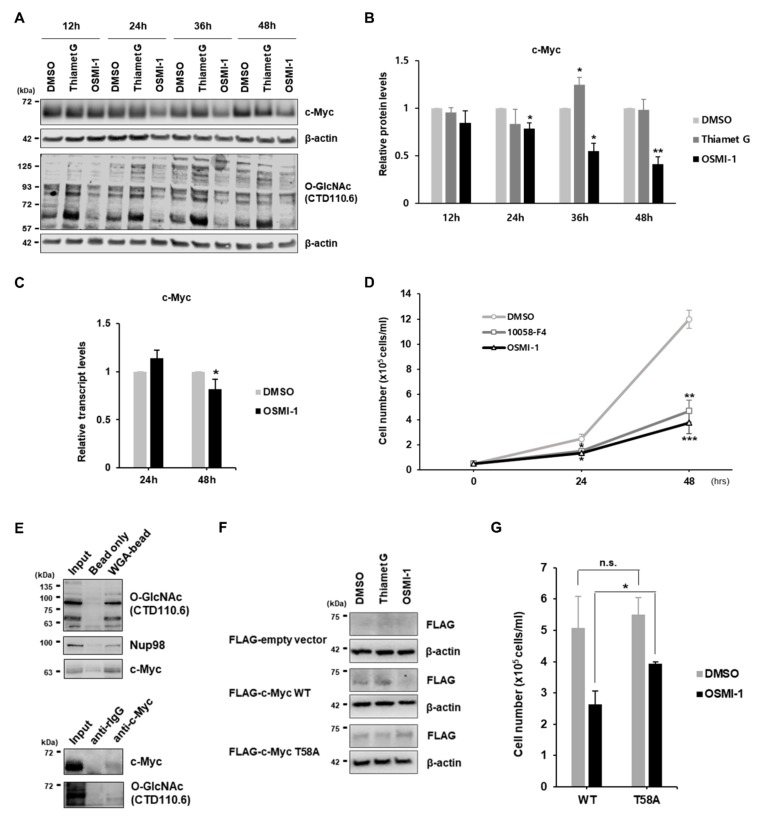
Stability of the c-Myc protein is substantially downregulated by inhibition of O-GlcNAcylation in pre-B cells. **(A**) Representative western blot to monitor changes in c-Myc protein levels and overall O-GlcNAcylation in PD36 pre-B cells cultured with inhibitors, Thiamet G (OGA inhibitor; 2 μM final) and OSMI-1 (OGT inhibitor; 10 μM final), for 48 h. (**B**) Quantification of c-Myc protein levels from the western blot in (**A**). Data are represented as mean ± SD (*n* = 3) normalized using β-actin as a loading control. Comparisons were performed using the DMSO control at each time point. (**C**) Real-time qRT-PCR analyses of changes in transcript levels of c-Myc in PD36 pre-B cells at 24 or 48 h post-treatment with OSMI-1 (10 μM, final). Data are represented as mean ± SD (*n* = 3) normalized using β-actin as a loading control. Comparison between OSMI-1 treatment and DMSO control. (**D**) Cell growth of PD36 pre-B cells for 48 h following inhibitor treatment (10058-F4 (Myc inhibitor; 50 μM final) and OSMI-1 (OGT inhibitor; 10 μM final)). The growth curve was generated by counting cells every 24 h. Data are represented as mean ± SD (*n* = 3). Comparisons were performed against the cell number in DMSO-treated control at each time point. (**E**) Western blot images, following pull-down using WGA-lectin conjugated to agarose beads (top) or immunoprecipitation of c-Myc proteins using an anti-c-Myc antibody with protein A-magnetic beads (bottom), to determine O-GlcNAc attachment to c-Myc proteins. (**F**) Representative western blot showing overexpressed protein levels of FLAG-c-Myc (WT or T58A) using the anti-FLAG antibody at 24 h post-treatment with Thiamet G (2 μM final) or OSMI-1 (10 μM final). (**G**) Cell growth of PD36 pre-B cells for 24 h under OSMI-1 (10 μM final) treatment following overexpression of FLAG-c-Myc (WT or T58A). Data are represented as mean ± SD (*n* = 3). Comparisons between WT and T58A were indicated. (**B**–**D**,**G**) Significance is annotated as * *p* ≤ 0.05, ** *p* ≤ 0.01, and *** *p* ≤ 0.001. n.s. = not significant.

To identify key molecules involved in regulating pre-B cell proliferation dependent upon cellular O-GlcNAc levels, we examined changes in the levels of several proteins involved in pre-B cell proliferation in the presence of O-GlcNAc inhibitors. Although signals associated with pre-BCR or Wnt/β-catenin activity are typically recognized as regulating pre-B cell proliferation [2,3,4,5], we found that the main components of both signaling pathways were expressed at normal levels under a low O-GlcNAcylation condition (Supplementary Figure S4A,B). However, levels of c-Myc (Myc proto-oncogene) protein, a potent regulator of cell proliferation [28,29], were significantly downregulated upon inhibition of OGT (Figure 3A,B) but not in the transcript levels (Figure 3C). Consequently, the growth of pre-B cells was also reduced to similar level as the treatment of OGT inhibitor when treated with Myc inhibitor 10058-F4 (Figure 3D). Because c-Myc is among the best-known O-GlcNAc target proteins, even in different cell types, and this modification is known to directly regulate c-Myc stability [36,37], we verified if c-Myc is modified by O-GlcNAcylation in pre-B cells as well and then determined its stability depending on the change of this modification. To isolate O-GlcNAc-attached proteins, we utilized a pull-down experiment using wheat germ agglutinin (WGA) lectins, which specifically bind to GlcNAc, conjugated with agarose beads. First, O-GlcNAcylated proteins were successfully confirmed by western blot, following pull-down, with an anti-CTD110.6 antibody as well as an anti-NUP98 antibody as positive controls (Figure 3E; top panel). Significantly, c-Myc proteins were also clearly detected in captured O-GlcNAcylated proteins from pre-B cell lysate using WGA-lectins compared to the ones with beads only as a negative control (Figure 3E; top panel). Conversely, O-GlcNAc attachment was also detected on the precipitated c-Myc proteins using an anti-c-Myc antibody from pre-B cell lysates according to western blot assay with an O-GlcNAc antibody (CTD110.6) (Figure 3E; bottom panel). These results show that c-Myc is directly O-GlcNAcylated in pre-B cells, similar to other cell types. A previous study showed regulation of c-Myc stability through competitive modification between phosphorylation and O-GlcNAcylation at the same site, threonine 58 (Thr58) [38], implying that c-Myc proteins might be stabilized by O-GlcNAcylation at Thr58 under upregulated O-GlcNAc conditions in large pre-B cells but destabilized by phosphorylation in the case of O-GlcNAc inhibition. In fact, the creation of the Thr58 mutation to alanine (T58A) in the FLAG-tagged construct of c-Myc wild-type cDNA resulted in increased c-Myc protein stability, regardless of O-GlcNAc inhibition by OSMI-1 treatment, relative to that observed in the wild-type construct (Figure 3F). However, c-Myc stability was not affected by thiamet G treatment, which increases O-GlcNAc levels (Figure 3F). In addition, pre-B cell proliferation, which was reduced by OSMI-1 treatment, was found to be partially recovered due to overexpression of c-Myc T58A compared to the wild type (Figure 3G). Collectively, these results indicate that c-Myc proteins in pre-B cells displaying increased O-GlcNAc levels are effectively stabilized through direct O-GlcNAcylation at Thr58, which induces cell proliferation.

### 3.4. O-GlcNAcylation and c-Myc Expressions are Regulated by Glucose Levels in Pre-B Cells

**Figure 4:**
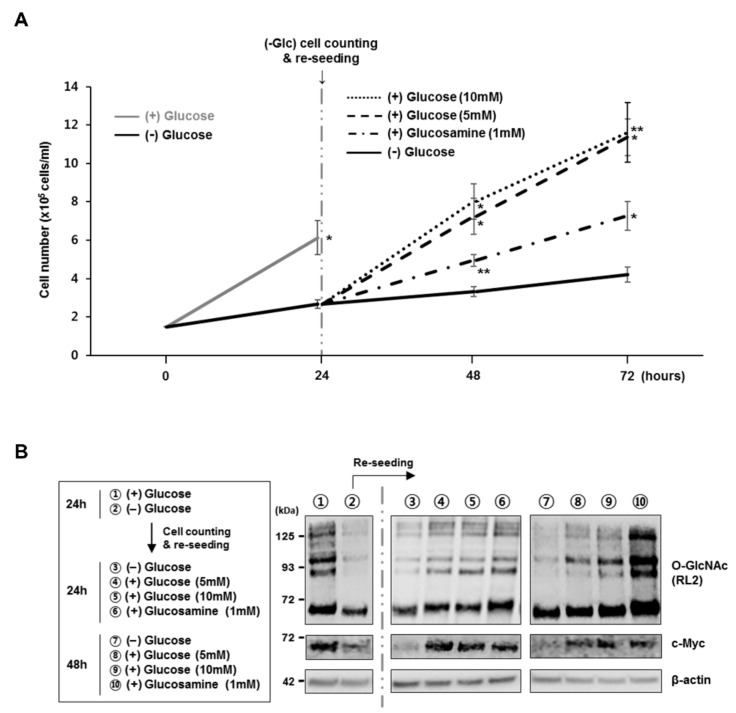
Decreased O-GlcNAc levels in pre-B cells due to the restriction of glucose uptake downregulates c-Myc expression and subsequently inhibits cell proliferation. (**A**,**B**) PD36 pre-B cells cultured in glucose-depleted media for the first 24 h were subsequently cultured following the re-introduction of glucose or glucosamine for another 48 h. (**A**) Cell growth in different culture conditions was monitored over 72 h. Data are represented as mean ± SD (*n* = 3). Comparisons were performed against the cell number in glucose-depleted media at each time point. Significance is annotated as * *p* ≤ 0.05, ** *p* ≤ 0.01. (**B**) The workflow of cell seeding and positions for western blot (left). Representative western blot showing changes in c-Myc protein levels and O-GlcNAcylation (right).

Because glucose or glucosamine are molecules necessary to produce UDP-GlcNAc, an OGT substrate, via the HBP, O-GlcNAc levels in cells are sensitive to the availability of these molecules. Thus, it was shown that depletion of glucose from culture media substantially reduced overall O-GlcNAc levels and significantly inhibited pre-B cell proliferation in the absence of cell death (Figure 4A,B; first 24 h). However, re-introduction of glucose or glucosamine to pre-B cells grown in glucose-depleted media for the first 24 h rescued cellular O-GlcNAc levels and recovered the attenuated growth rate relative to control cells continuously cultured without glucose (Figure 4A,B; post-24 h). Interestingly, while cellular O-GlcNAc levels were dynamically changed depending on glucose availability, c-Myc levels were also consequently altered (Figure 4B). Indeed, downregulated c-Myc levels in the presence of low O-GlcNAcylation via glucose restriction were completely recovered by upregulated O-GlcNAcylation following the re-introduction of glucose or glucosamine in the culture media. These results further confirm that pre-B cell proliferation is tightly dependent on the stability of c-Myc proteins, which is controlled by cellular O-GlcNAc levels according to glucose availability.

### 3.5. Decreased O-GlcNAcylation in Pre-B Cells Results in Cell Cycle Arrest via Suppression of Cyclin Expressions

**Figure 5:**
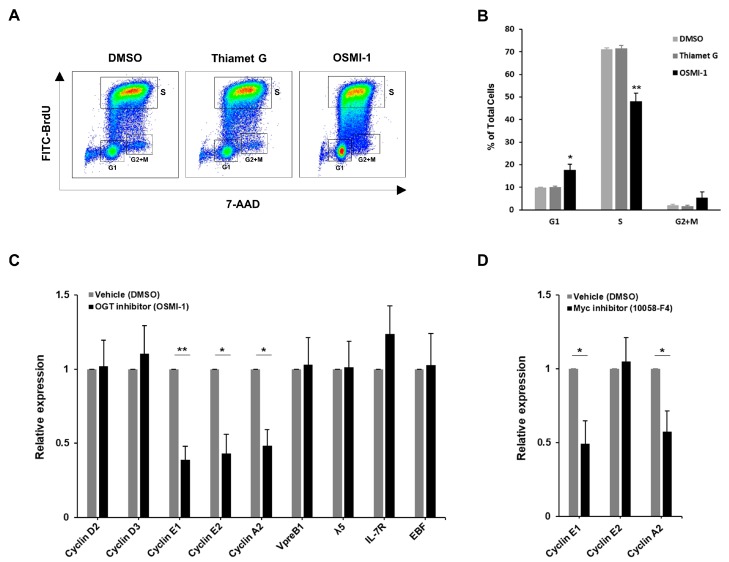
Inhibition of O-GlcNAcylation in pre-B cells causes cell cycle arrest through suppression of E- and A-type cyclin expression. (**A**) Representative plot showing data from flow cytometric analysis of cell cycle progression in PD36 pre-B cells double-stained with 7-AAD and BrdU-FITC at 48 h post-treatment with inhibitors [Thiamet G (2 μM, final) and OSMI-1 (10 μM, final)]. (**B**) Quantification of the percentage of cells at each cell cycle stage derived from (**A**). Data are represented as mean ± SD (*n* = 3). Comparison between OSMI-1 treatment and DMSO control at each stage. (**C**,**D**) Real-time qRT-PCR analyses of changes in the transcript levels for each target gene in PD36 pre-B cells at 48 h post-treatment with 10 μM of OSMI-1 (**C**) and 50 μM of 10058-F4 (**D**). Data are represented as mean ± SD (*n* = 3) normalized using β-actin as a loading control. Comparisons were performed against the DMSO control for each target gene. (**B**–**D**) Significance is annotated as * *p* ≤ 0.05, ** *p* ≤ 0.01.

Reduced cell proliferation by altered c-Myc levels is generally attributed to cell cycle arrest via the downregulated expression of cyclins or the upregulated expression of cyclin kinase inhibitors, such as p21 or p27 [28,29]. To confirm this under our experimental conditions involving altered O-GlcNAc levels, we first analyzed cell cycle progression in pre-B cells by combinatorial staining of the total DNA content (7-aminoactinomycin D, 7-AAD) and newly synthesized DNA with fluorescein isothiocyanate (FITC) conjugated to bromodeoxyuridine (BrdU). Pre-B cells treated with an OGT inhibitor showed cell cycle delays resulting from severe increases in the number of cells in the G1 phase, as well as decreases in those in the S-phase, as compared with normal cell cycle progression observed in vehicle-control- or OGA-inhibitor-treated cells (Figure 5A,B). However, monocytic THP-1 cells showing normal cell growth under a low O-GlcNAc condition did not reveal any changes in their cell cycle status (Supplementary Figure S3C). Functionally, expression of E- and A-type cyclins significantly decreased under a low O-GlcNAc condition in pre-B cells (Figure 5C), whereas that of cyclin kinase inhibitors, like p21 or p27, was unaffected (Supplementary Figure S4C). Additionally, we further confirmed that the transcript levels of other key factors associated with pre-BCR or IL-7R signaling, involved in pre-B cell proliferation [2,3,6], were utterly unaffected by changes in O-GlcNAc levels (Figure 5C). Interestingly, Myc inhibitor treatment also reduced cyclin E1 and A2 expressions similar to that of OGT inhibition, but not E2, which means cyclin E2 may not be contained within the signaling system to which O-GlcNAcylation and c-Myc are connected (Figure 5D). In addition to the known knowledge of the role of c-Myc in regulating cyclin expression [39], our findings suggest that downregulated c-Myc levels via inhibited O-GlcNAcylation in pre-B cells may directly suppress the expression of selected cyclins, like E1 or A2.

### 3.6. Inhibition of O-GlcNAcylation in Pre-B Cells by OGT Knockdown Decreases Cell Proliferation as well as c-Myc Expression

**Figure 6:**
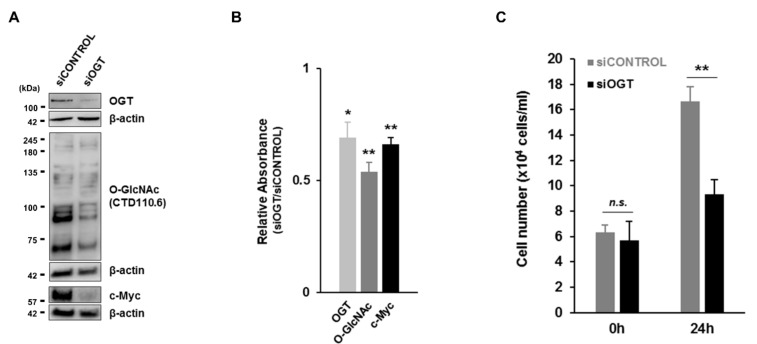
Suppressed O-GlcNAcylation in pre-B cells by OGT knockdown causes decreased cell proliferation via reduction of c-Myc expression. (**A**) Representative western blot to monitor changes in OGT, c-Myc, and overall O-GlcNAc levels in PD36 pre-B cells transfected with siOGT for 24 h compared to siCONTROL. (**B**) Relative absorbance measured using ELISA for total OGT (0.125 mg/mL), c-Myc (0.125 mg/mL), and O-GlcNAc (4 μg/mL) protein levels in different concentrations of lysate of PD36 pre-B cell transfected with siOGT for 24 h compared to siCONTROL. Data are represented as mean ± SD (*n* = 3). Comparisons between siCONTROL and siOGT. (**C**) Cell growth with knock-down of OGT was checked at 24 h compared to control cells. Data are represented as mean ± SD (*n* = 3). Comparisons were performed for cell numbers between siCONTROL and siOGT. (**B**,**C**) Significance is annotated as * *p* ≤ 0.05, ** *p* ≤ 0.01. n.s. = not significant.

To verify our results through the specific inhibition of OGT expression, we utilized an OGT knockdown experiment on pre-B cell proliferation. First, OGT expression was effectively inhibited in pre-B cells transfected with siRNA for OGT (siOGT) compared to scrambled siRNA (siCONTROL) as a negative control as confirmed in the western blot assay (Figure 6A) or a semi-quantitative ELISA (Figure 6B). Under this condition, overall O-GlcNAc levels were reduced, and significantly, c-Myc protein levels also accordingly decreased as similarly shown in the treatment with an OGT inhibitor to pre-B cells (Figure 6A,B). In addition, the proliferation of pre-B cells under OGT knockdown was much slower than control cells transfected by siCONTROL (Figure 6C). Thus, these results more specifically support that the stability of c-Myc proteins is tightly regulated through its O-GlcNAc modification by OGT, which is a critical operator in controlling the proliferation of pre-B cells.

### 3.7. Administration of an OGT Inhibitor Negatively Affects the Formation of the Normal B Cell Population in the Bone Marrow of Mice

**Figure 7:**
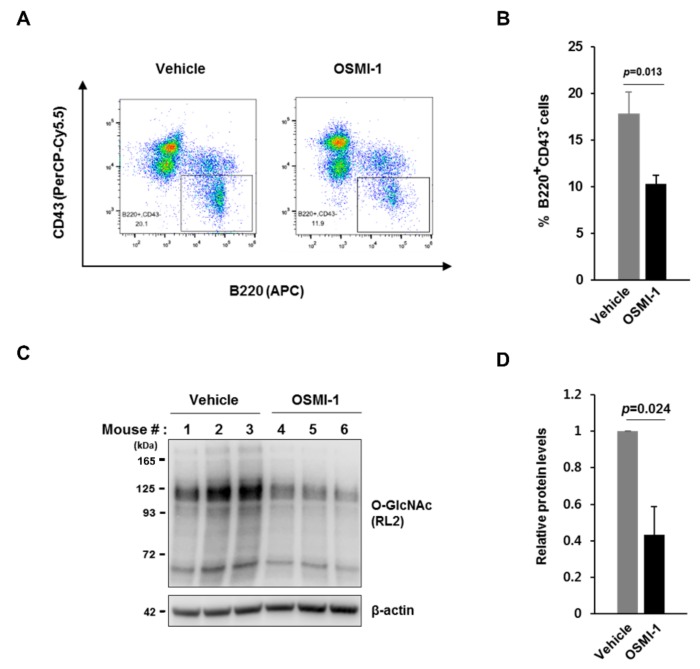
The population of B cells after the pro-B cell stage in the bone marrow is markedly reduced by administration of an OGT inhibitor to mice. (**A**) Representative plot of the flow cytometric analysis data, including PerCP-Cy5.5-CD43 and APC-B220 staining of total bone marrow (BM) cells from mice at 7 days after daily injection of OSMI-1 or a vehicle control. (**B**) Quantification of the percentage of B220^+^CD43^−^ cells from (**A**). Data are represented as mean ± SD (*n* = 3). Comparison of OSMI-1 treatment with the vehicle control. (**C**) Western blot analysis of changes in O-GlcNAcylation in BM cells from mice at 7 days after daily injection of OSMI-1 or the vehicle control. (**D**) Quantification of relative protein levels according to western blot results from (**C**). Data are represented as mean ± SD (*n* = 3) normalized using β-actin as a loading control. Comparison of OSMI-1 treatment with the vehicle control.

We then determined whether inhibition of O-GlcNAc levels in mouse bone marrow would influence the normal B cell population via the change of pre-B cell growth. We first established a preparation method for the transfer of the OGT inhibitor OSMI-1 in vivo, using 1 mg of OSMI-1 solubilized in 100 μL of buffer containing 4.5% DMSO and 5% Tween-80 in water. We then injected this solution intraperitoneally every 24 h for 1 week into 5-week-old female BALB/c mice immunized once with NP-CGG in alum on the second day. The next day, following the final OSMI-1 injection, we extracted total bone marrow cells and subjected them to FACS analyses using CD43 and B220 as markers to distinguish between pro-B and other B cells after the pro-B stage. As expected, the total B cell population after the pro-B cell stage in the bone marrow from mice injected with the OGT inhibitor was significantly reduced as compared with those from vehicle controls (injection of buffer only) (Figure 7A,B) in the absence of differences in body weight between inhibitor-injected mice and controls (data not shown). Critically, our OGT inhibitor injection strategy was highly effective, given the observed inhibition of overall O-GlcNAc levels in total bone marrow cells from injected mice (Figure 7C,D). These findings suggest that the suppressed condition of O-GlcNAcylation in mouse bone marrow by the administration of an OGT inhibitor impedes the proper expansion of functional B cell clones, presumably resulting from the restriction of large pre-B cell proliferation, during early B cell development.

## 4. Discussion

Levels of the c-Myc proto-oncogene are important not only for the development of B cell-derived cancers but also for normal B cell proliferation because c-Myc protein is strongly linked to cell cycle progression [40]. To avoid lymphomagenesis, c-Myc expression in normal B cells is tightly regulated at multiple levels [41]. For example, c-Myc transcription is flexibly regulated at the elongation stage through differential control of the engagement of RNA polymerase II complexes on the c-Myc transcription-start site by growth factors (positive regulation) or differentiation-related factors (negative regulation). Similar to the rapid turnover of other cell cycle regulators, the half-life of c-Myc mRNA and protein is also limited to 30 min in resting B cells, although mRNA stability can be increased to several hours in activated B cells, and protein stability can be altered by posttranslational modifications during the course of B cell development. Therefore, c-Myc expression is tightly and dynamically regulated in B cells, depending on their differentiation or activation status. Nevertheless, a common aspect of many B cell-derived cancers still continues to be the constitutive overexpression of c-Myc. One reason for this dysregulation in lymphomas is genetic rearrangement caused mainly by reciprocal translocation of c-Myc into the proximity of Ig genes [40]. However, several acute lymphoblastic leukemias (ALLs) derived from B cell precursors do not carry these genetic lesions, despite the presence of c-Myc overexpression [42], implying the existence of other mechanisms controlling dysregulated c-Myc expression. Apart from B cell tumors, little is known about the upstream mechanisms linked to c-Myc expression, and that also regulate normal pre-B cell proliferation. In this study, we found that O-GlcNAcylation was significantly induced at the proliferative pre-B cell stage during early B cell development, which stabilized c-Myc expression by inducing O-GlcNAcylation, and enabled the rapid proliferation of functional B cell clones expressing pre-BCR. Because these findings were verified not only in vitro but also in vivo, our findings suggest O-GlcNAcylation as a new mechanism for the regulation of c-Myc expression during normal pre-B cell proliferation, as well as potentially in pre-B cell-derived ALL.

Similar to phosphorylation, O-GlcNAcylation can dynamically change the function of its downstream target proteins. However, phosphorylation is dependent on hundreds of kinases and phosphatases, whereas cellular O-GlcNAcylation is modulated by only two enzymes (OGT and OGA), suggesting that a wide range of proteins in cells can be affected by intentional inhibition of these two enzymes. Searching for key proteins involved in the suppression of pre-B cell growth under OGT inhibition, we first expected to find alterations in O-GlcNAc target proteins, other than c-Myc, such as β-catenin and glycogen synthase kinase (GSK)3β in the Wnt/β-catenin pathway, because both were confirmed as O-GlcNAc target proteins in different cell systems [43] and are also important for pre-B cell proliferation. However, low O-GlcNAcylation conditions had little influence on β-catenin and GSK3β activities (Supplementary Figure S4). This result might be explained by the heterogeneous effect of O-GlcNAc inhibition. Previous studies showed that the catalytic activities of both enzymes vary widely depending on each target protein [44]. Additionally, the effects of OGT or OGA inhibition are cell type-dependent [45]. Indeed, OGT or OGA modify their target proteins in a cell- or tissue-specific and protein-specific manner. Although comparative and quantitative analyses are required to precisely distinguish the proteins and/or genes altered under low O-GlcNAcylation conditions, we showed that c-Myc were more sensitive targets of OGT in pre-B cells than β-catenin or GSK3β.

We confirmed that the administration of an OGT inhibitor to mice significantly reduced the total B cell population after the pro-B cell stage in the bone marrow. However, Wu et al. [25] showed that conditional *Ogt* knockout in B cells affected only the number of mature B cells but not other stages of B cell development in the bone marrow. This implies that the absence of OGT does not influence normal B cell development in the bone marrow, as most mature B cells in bone marrow constitute re-circulating B cells from secondary lymphoid organs. This result contradicts our observation of a reduced B cell population following OGT inhibition in mouse bone marrow. Although a knockout study would be more convincing, a reasonable elucidation is essential to explain our observation. First, we evaluated changes in the B cell population under immunization because it was expected that pre-existing B cells in the bone marrow of immunized mice may be more quickly egressed to secondary lymphoid organs than in the normal mouse, and then, the inhibitory effect on pre-B cell expansion after injecting the inhibitor could be more clearly shown, whereas Wu et al. [25] compared the B cell population between normal and knock-out mice without any immunization. In fact, relatively smaller changes showing only a <20% decrease in the B cell population by inhibitor injection were observed in non-immunized mice in our hands, as well. Second and more significantly, knockout mice often establish compensatory strategies to promote normal growth or differentiation. For example, a previous study reported that B cell development was not completely blocked in surrogate-light-chain-deficient mice, established via functional replacement of a conventional BCR combined with Igμ and IgL chains instead of elimination of pre-BCR, thereby allowing developmental rescue [2]. Therefore, we assumed that *Ogt* conditional knockout mice studied in Wu et al. should be inherently challenged in their ability to expand large pre-B cell clones through the O-GlcNAc pathway, and presumably, other independent mechanisms not regulated by O-GlcNAc likely compensated for the attenuated OGT function in pre-B cell proliferation in order to maintain adequate levels of B cell development.

## 5. Conclusions

In this study, we showed that the proliferation of large pre-B cells was triggered by induction of O-GlcNAcylation during the transition from pro-B to pre-B cells during early B cell development. Significantly, c-Myc expression was directly regulated by O-GlcNAcylation, which, in turn, controlled pre-B cell proliferation. Given the positive correlation between cancer development and c-Myc overexpression, as well as O-GlcNAcylation, O-GlcNAcylation-dependent induction of c-Myc expression at the pre-B cell stage should be restricted thoroughly to prevent uncontrolled proliferation. Paradoxically, the fine regulation of O-GlcNAcylation might represent a promising therapeutic target for the treatment of B cell-precursor-derived leukemia.

## Supplementary Materials

The following are available online at https://www.mdpi.com/2073-4409/9/1/158/s1, Figure S1: Pro-B cells and large pre-B cells were sequentially sorted by MACS using B220 microbeads and then by FACS using CD43 and IgM or pre-BCR as markers, Figure S2: Pre-B cell proliferation is significantly decreased by inhibition of O-GlcNAcylation without any significant cell death, Figure S3: THP-1 cell growth is unaffected by treatments of O-GlcNAc related inhibitors, Figure S4: Unchanged expression of factors regulating pre-B cell proliferation following inhibitor treatment, Table S1: Primers used in this study.
