# Supplementary material for: Increased O-GlcNAcylation of c-Myc Promotes Pre-B Cell Proliferation
Source: Cells. 2020 Jan 8;9(1):158. doi: 10.3390/cells9010158 (PMC7016991; doi:10.3390/cells9010158)
Supplement: Supplementary file 1 [file cells-09-00158-s001.pdf]

**Supplementary Material**

# Increased O-GlcNAcylation of c-Myc Promotes Pre-B Cell Proliferation

*Da Hee Lee[1], Na Eun Kwon[1], Won-Ji Lee[2], Moo-Seung Lee[3], Doo-Jin Kim[1], Ji-Hyung Kim[1], and Sung-Kyun Park[1,*]*

*[1]Infectious Disease Research Center, Korea Research Institute of Bioscience & Biotechnology (KRIBB), 125 Gwahak-ro, Daejeon 34141, Republic of Korea*

*[2]Department of Stem Cell and Regenerative Biotechnology, Konkuk Institute of Technology, Konkuk University, Seoul 05029, Republic of Korea*

*[3]Environmental Diseases Research Center, Korea Research Institute of Bioscience & Biotechnology (KRIBB), 125 Gwahak-ro, Daejeon 34141, Republic of Korea*

***Correspondence**: Sung-Kyun Park, skpark@kribb.re.kr*

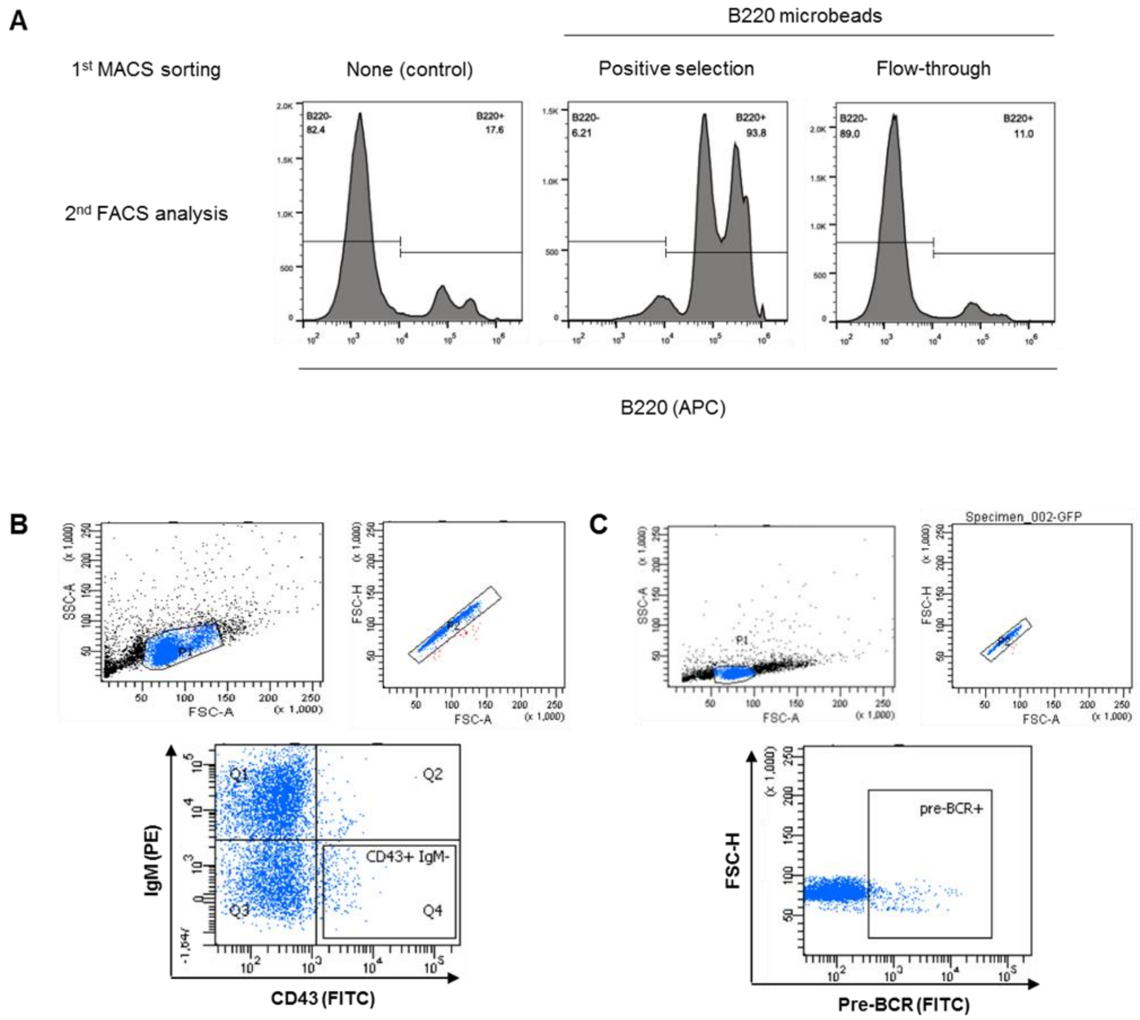


**Figure S1. Pro-B cells and large pre-B cells were sequentially sorted by MACS using B220 microbeads and then by FACS using CD43 and IgM or pre-BCR as markers**

(A) Representative plot of the FACS analysis using B220-APC shows the purity of B220-microbeads positive cells from mouse bone marrow sorted by MACS. (B) Pro-B cells ($CD43^{+}IgM^{-}$) were isolated by FACS using CD43-FITC and IgM-PE after B220 enrichment by MACS. (C) Large pre-B cells were pre-BCR-$FITC^{+}$ sorted by FACS after B220 enrichment by MACS.

**A**

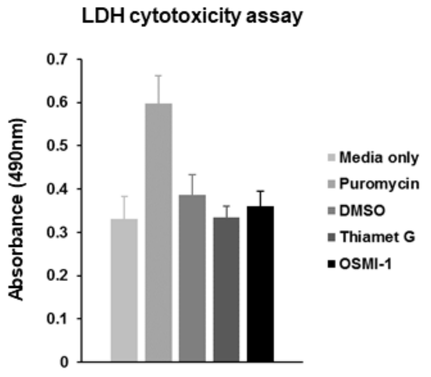


**B**

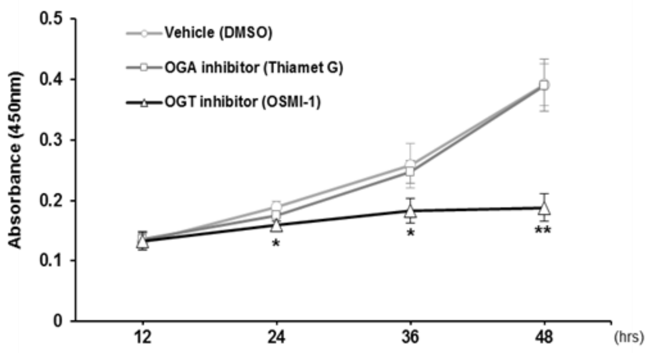


**Figure S2. Pre-B cell proliferation is significantly decreased by inhibition of O-GlcNAcylation without any significant cell death**

(A) LDH cytotoxicity assay performed after a 48-h culture following inhibitor treatment [Thiamet G (2 μM, final) and OSMI-1 (10 μM, final)]. Cells were incubated with puromycin (10 μg/mL) for 5 h to artificially induce cell damage as a positive control, resulting in a ~35% death rate. Background levels of LDH activity were established using culture medium alone. Data are represented as mean ± SD (n = 3). Comparison of each treatment with medium alone control. n.s. = not significant. (B) WST-1 dye-based cell-proliferation assay. The growth of PD36 pre-B cells was monitored for 48 h following inhibitor treatment [Thiamet G (OGA inhibitor; 2 μM, final) and OSMI-1 (OGT inhibitor; 10 μM, final)]. Data represent the mean ± SD (*n* = 3). Comparison of OSMI-1 treatment with DMSO control at each time point.

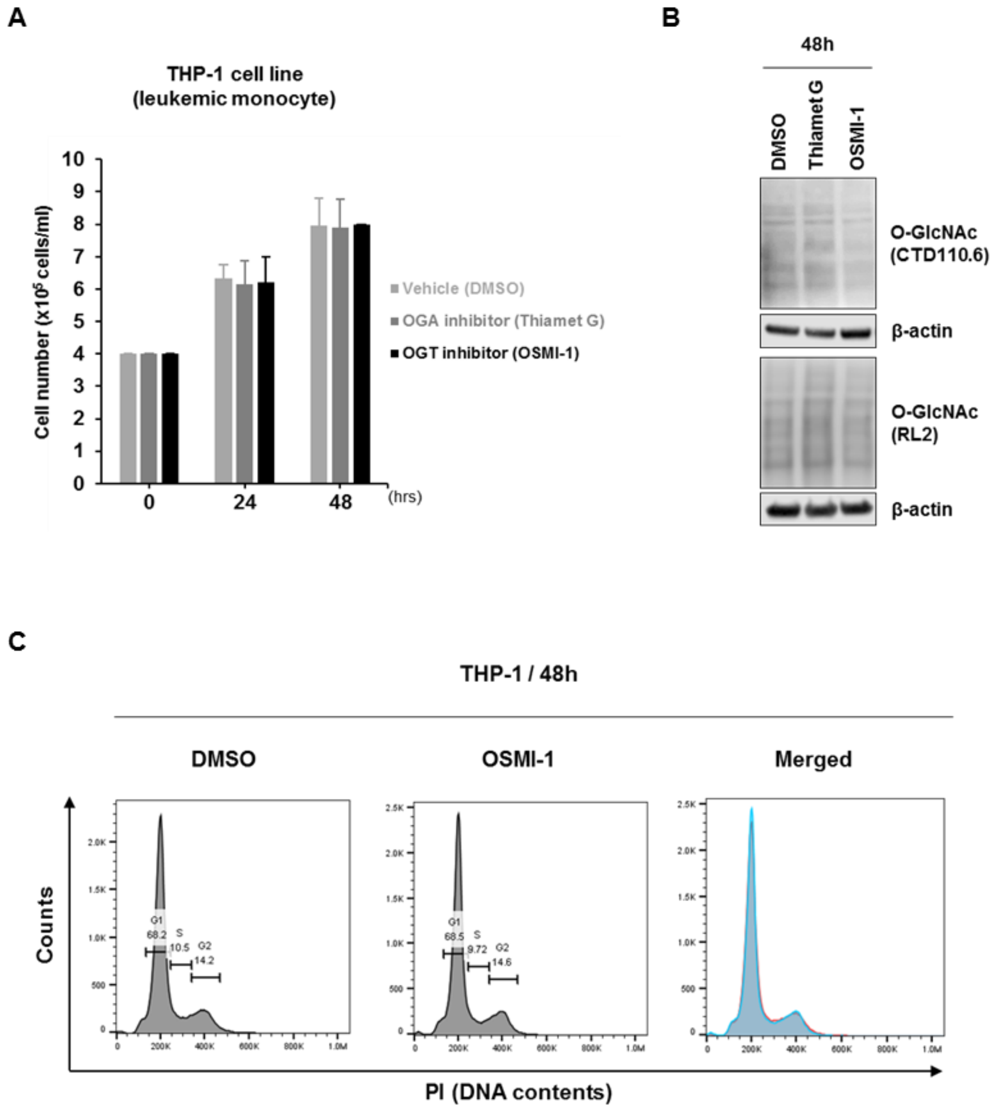


**Figure S3. THP-1 cell growth is unaffected by treatments of O-GlcNAc related inhibitors**

(A) The growth of THP-1 cells was monitored for 48 h following inhibitor treatment [Thiamet G (2 μM, final) and OSMI-1 (10 μM, final)]. Data represent the mean ± SD ($n$ = 3). (B) Representative western blot monitoring changes in O-GlcNAcylation levels. (C) Representative plot of the flow cytometric analysis of THP-1 cell cycle progression following staining with propidium iodide (PI) at 48 h after treatment with OSMI-1 (10 μM, final). The percentages of cells at the G1, S, and G2/M phases are shown. Merged plots revealed no change in the cell cycle stage according to treatment.

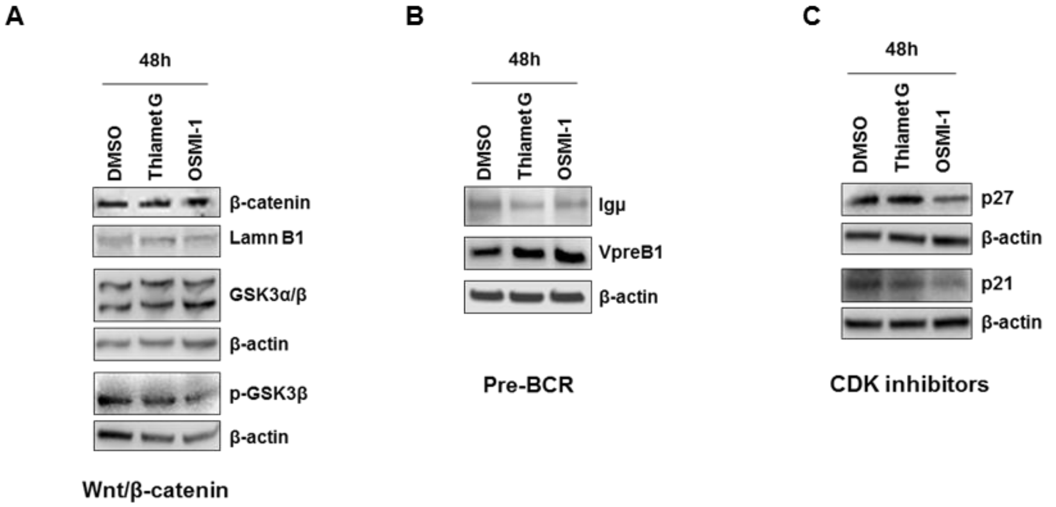


**Figure S4. Unchanged expression of factors regulating pre-B cell proliferation following inhibitor treatment**

(A-C) Representative western blot monitoring protein levels associated with Wnt/β-catenin signaling (A), pre-BCR signaling (B), or CDK inhibitors like p21 and p27 (C) at 48-h post-treatment with inhibitors [Thiamet G (2 μM, final) and OSMI-1 (10 μM, final)].

| Name | Description | Use | Sequence(5'→3') |
|---|---|---|---|
| SP042 | FOR for Cyclin D2 qPCR (mouse) | qPCR | GGA CAT CCA ACC GTA CAT G |
| SP043 | REV for Cyclin D2 qPCR (mouse) | qPCR | GAA AGA CCT CTT CTT CAC AC |
| SP083 | FOR for Cyclin D3 qPCR (mouse) | qPCR | GCT TTG CAT CTA TAC GGA CC |
| SP084 | REV for Cyclin D3 qPCR (mouse) | qPCR | GAA TCA AGG CCA GGA AGT C |
| SP099 | FOR for Cyclin E1 qPCR (mouse) | qPCR | CCGTGTTTTTGCAAGACCCAG |
| SP100 | REV for Cyclin E1 qPCR (mouse) | qPCR | GGTCTGATTTTCCGAGGCTG |
| SP069 | FOR for Cyclin E2 qPCR (mouse) | qPCR | CAC CCC ATA AAG AAA TAG GAA CAA G |
| SP070 | REV for Cyclin E2 qPCR (mouse) | qPCR | CCC AGC TTA AAT CTG GCA GAG |
| SP087 | FOR for Cyclin A2 qPCR (mouse) | qPCR | CTC AAG ACT CGA CGG/ GTT |
| SP088 | REV for Cyclin A2 qPCR (mouse) | qPCR | CTC CAT TTC CCT AAG GTA CG |
| SP101 | FOR for VpreB qPCR (mouse) | qPCR | GCCCACCTCACAGGTTGT |
| SP102 | REV for VpreB qPCR (mouse) | qPCR | CCTGGCCGTATCTTTGGACC |
| SP005 | FOR for λ5 qPCR (mouse) | qPCR | CTT GAG GGT CAA TGA AGC TCA GAA GA |
| SP006 | REV for λ5 qPCR (mouse) | qPCR | CTT GGG CTG ACC TAG GAT TG |
| SP052 | FOR for IL-7Rα qPCR (mouse) | qPCR | GCC CAG CAA GGG GTG AAA GCA AC |
| SP053 | REV for IL-7Rα qPCR (mouse) | qPCR | GGC AAG ACA GGA TCC CAT CCT CC |
| SP007 | FOR for EBF qPCR (mouse) | qPCR | GCT GTG GCA ACC GAA ATG AG |
| SP008 | REV for EBF qPCR (mouse) | qPCR | CCG TGC TTG GAG TTA TTG TGG AC |
| SP119 | FOR for c-Myc_T58A | site-directed mutagenesis | CCCGCCCCGCCCCTGTCCCCG |
| SP120 | REV for c-Myc | site-directed mutagenesis | AAGCAGCTCGAATTTCTTCCA |

**Table S1. Primers used in this study**
